# Unusual “Asian-origin” 2c to 2b point mutant canine parvovirus (*Parvoviridae*) and canine astrovirus (*Astroviridae*) co-infection detected in vaccinated dogs with an outbreak of severe haemorrhagic gastroenteritis with high mortality rate in Hungary

**DOI:** 10.1007/s11259-022-09997-2

**Published:** 2022-09-21

**Authors:** Ákos Boros, Mihály Albert, Péter Urbán, Róbert Herczeg, Gábor Gáspár, Benigna Balázs, Attila Cságola, Péter Pankovics, Attila Gyenesei, Gábor Reuter

**Affiliations:** 1 Department of Medical Microbiology and Immunology, Medical School, University of Pécs, Pécs, Hungary; 2 Ceva Phylaxia Ltd. Budapest, Budapest, Hungary; 3 Szentágothai Research Centre, Bioinformatics Research Group, Genomics and Bioinformatics Core Facility, University of Pécs, Pécs, Hungary

**Keywords:** Canine Parvovirus 2, Canine astrovirus, Co-infection, Cultivation, NGS, Gastroenteritis

## Abstract

In this study, the aetiological background of an outbreak of severe haemorrhagic gastroenteritis (HGE) in a colony of purebred Jack Russell Terriers vaccinated against CPV-2 in Hungary was investigated. Canine parvovirus 2 (CPV-2, *Parvoviridae*) and canine astrovirus (CaAstV, *Astroviridae*) co-infection was identified by viral metagenomics and next-generation sequencing (VM-NGS) methods from a rectal swab of an affected 7-week-old puppy. The complete coding sequence of CPV-2 strain FR1/CPV2-2021-HUN (ON733252) and the complete genome of CaAstV strain FR1/CaAstV-2021-HUN (ON733251) were determined by VM-NGS and PCR methods. Results of sequence and phylogenetic analyses showed that CPV-2 strain FR1/CPV2-2021-HUN was different from the applied vaccine strains and previously identified strains from Hungary but showed high sequence identity (> 99.8%) and close phylogenetic relationship to recently described “Asian-origin” CPV-2c strains from Italy. But, based on the single amino acid difference on position 426 of VP2 (Glu/Asp) between the study strain and the closest relatives, FR1/CPV2-2021-HUN belonged to the 2b antigenic type rather than 2c. The CaAstV strain FR1/CaAstV-2021-HUN showed close relationship with a CaAstV strain identified previously from a diarrhoeic dog in Hungary. Both viruses were continuously detectable by PCR in additional enteric samples, and the CPV-2 could also be detected in several (*n* = 32) tissue samples from 9 affected deceased puppies. Further comparative studies are necessary to confirm the role of the point mutation causing the change in the antigenic type of this “Asian-origin” CPV-2 and/or the role of CaAstV co-infection in the development and/or severity of (haemorrhagic) gastroenteritis among dogs vaccinated against CPV-2.

**Supplementary Information:**

The online version contains supplementary material available at 10.1007/s11259-022-09997-2.

## Introduction

*Canine parvovirus type 2* (CPV-2) together with its ancestor feline panleukopenia virus are currently classified into the family *Parvoviridae,* genus *Protoparvovirus,* species *Carnivore protoparvovirus 1* (Cotmore et al. 2019). The currently circulating variants of CPV-2 may cause outbreaks of severe haemorrhagic gastroenteritis (HGE) with a high (up to 70%) mortality rate among unvaccinated susceptible dogs (Decaro and Buonavoglia 2012). The clinical signs of disseminated CPV-2 infections are mostly characterized by vomiting, mucoid/bloody diarrhoea and acute lymphopenia in young puppies, although subclinical infections are also being observed typically in adult dogs (Decaro and Buonavoglia 2012).

CPV-2 is a small, non-enveloped virus with an approximately 5.2 kb-long linear, negative-sense, single-stranded DNA genome, which contains two major non-overlapping open reading frames (ORFs) flanked by 3’ and 5’ untranslated regions (UTRs). The UTRs contain multiple repeated/duplicated sequence motifs which could form stable hairpin structures. The first ORF encodes non-structural proteins (NS1 and its splice variant NS2), and the other encodes capsid proteins (VP1 and its truncated version VP2) (Reed et al. 1988; Decaro and Buonavoglia 2012; Tuteja et al. 2022) . VP2 is the major capsid protein which contains the main antigenic sites (Tsao et al. 1991; Qi et al. 2020). Based on the presence of Asparagine (official one-letter code: N), Aspartic acid (D) or Glutamic acid (E) amino acids (aa) on position 426 of VP2 the CPV-2 strains could be categorized into main antigenic variants 2a, 2b or 2c, respectively; although the VP2_426_-based typing does not always reflect the true phylogenetic relationship of CPV-2 viruses (Decaro and Buonavoglia 2012; Qi et al. 2020).

Different variants of CPV-2 are presented worldwide with variable geographic distribution and abundance (Miranda and Thompson, 2016). Nowadays the rapid spread of recently emerged “Asian-origin” CPV-2c strains with characteristic aa mutations of NS1 (**60 V**, **544F**, **545 V**, **630P);** NS2 (**60 V**, **151 N**, **152 V)** and VP2 (**5G**, **267Y**, **297A**, **324I** and **370R**) are causing serious concerns in Asia and Europe as well (Mira et al. 2019; Novosel et al. 2019; Balboni et al. 2021; Chen et al. 2021). There are multiple types of commercially available and widely used vaccines against CPV-2, although still several CPV-2 infections-related HGE cases have been documented among vaccinated dogs (Decaro et al. 2008; Qi et al. 2020).

Co-infections of CPV-2 with other pathogens are frequently found but tested only occasionally. If investigated at all, mostly CPV-2 variants or other gastroenteritis (GE)-associated agents have been tested, mostly with conventional PCR techniques (Miranda and Thompson, 2016; Qi et al. 2020). Furthermore, the specific detection/discrimination of vaccine and/or wild-type strains presented in the faeces of HGE cases could be a diagnostic challenge in PCR-based techniques (Decaro and Buonavoglia 2012; Tuteja et al. 2022) .

Next-generation sequencing (NGS) techniques could serve as an effective tool for the genome-based detection, discrimination and sequence analyses of multiple types of pathogens in various sample types including faeces. Besides CPV-2, multiple other enteric viruses were identified from faeces of diarrhoeic dogs including canine astroviruses (CaAstVs) in family *Astroviridae* (Mihalov-Kovács et al. 2017) or canine sapovirus of family *Caliciviridae* (Stamelou et al. 2022). CaAstVs have a poly (A)-tailed, positive sense, single-stranded RNA genome with two overlapping ORFs (ORF1ab and ORF2). CaAstVs could be identified frequently from enteric samples of both asymptomatic and diarrhoeic dogs, sometimes as a co-infecting agent with other enteric pathogens (Mihalov-Kovács et al. 2017; Bhatta et al. 2019).

In this study the possible aetiological background of an outbreak of acute haemorrhagic gastroenteritis among 7–8 weeks-old purebred Jack Russel terriers vaccinated freshly against CPV-2 was investigated using unbiased viral metagenomics, NGS and various PCR methods.

## Materials & methods

A recurrent outbreak with typical signs of CPV-2-associated parvovirosis including (fever, anorexia, lethargy, bloody-foamy diarrhoea and vomiting) was present in the colony of approximately 20 bitches and their offspring between March of 2021 and January of 2022. The manifested disease was affecting only 7–8 weeks-old puppies with a high (≈60%) mortality rate. Initially, the puppies received vaccines of either Canigen® DHPPi (VirBac, Carros, France) or Primodog™ (Boehringer Ingelheim Animal Health, Ingelheim am Rhein, Germany) which contains live, attenuated CPV-2b strains of Cornell-780916. The first dose was given subcutaneously at 6 weeks of age followed by a second vaccination 2–3 weeks later based on the instructions of the manufacturers. Due to the manifestation of HGE in vaccinated dogs few days after receiving the first dose, the immunization schedule was altered as follows: the first dose was given at 3 weeks of age of all subsequently born puppies followed by a second vaccination 2 weeks later, but the disease and deaths were still present. Therefore, investigation was started to identify the potential aetiological reasons behind the disease. Multiple rectal swabs and additional samples of various organs were collected from naturally deceased animals for further investigations (Table 1). Previous histological examinations of small intestine from the deceased animals showed the signs of villous atrophy, necrosis of crypts and nuclear inclusion bodies (data not shown). Previous laboratory tests showed no signs of *Salmonella* spp., *Campylobacter* spp., *Yersinia* spp., Canid herpesvirus 1 (CHV-1) or Canine coronavirus (CCoV) in the enteric samples of affected puppies.

**Table 1:** Origin and features of dogs and samples used in this study as well as data of canine parvovirus 2 (CPV-2) and canine astrovirus (CaAstV) PCR positivity. Tissue samples originating from naturally deceased puppies showed the signs of haemorrhagic gastroenteritis (HGE). The original sample used for viral metagenomics and next-generation sequencing was underlined. For the oligonucleotide primer pairs used for CPV-2 and CaAstV screening reverse-transcription-PCR (RT-PCR) and conventional PCR (cPCR) reactions and technical details see Table S1 and Online Resource. + : PCR-positive sample, − : PCR-negative sample. # Positivity was confirmed by Sanger-sequencing of the PCR-product

Dog ID	Age	Gender	Symptoms	Sampling Date	Sample Type	CPV-2 cPCR	CaAstV RT-PCR
FR1	7 weeks	male	HGE (deceased)	02.07.2021	rectal swab	+ #	+ #
					intestine	+	+
					spleen	+ #	−
					liver	−	−
					lymph node	+	−
JR1	7 weeks	female	HGE (deceased)	12.07.2021	rectal swab	+ #	+ #
					intestine	+	+ #
					lymph node	+ #	−
					spleen	+	−
					thymus	+ #	−
					bone marrow	−	−
JR2	7 weeks	male	HGE (deceased)	12.07.2021	rectal swab	+ #	+ #
					intestine	+	+
					lymph node	+	−
					spleen	+	−
					thymus	+ #	−
					bone marrow	+	−
JR3	8 weeks	female	HGE (deceased)	18.09.2021	rectal swab	+ #	+ #
					intestine	+	+ #
					bone marrow	−	−
					spleen	+ #	−
					lymph node	+	−
					thymus	+ #	−
JR4	8 weeks	male	HGE (deceased)	18.09.2021	rectal swab	+ #	+ #
					intestine	+	+
					bone marrow	+ #	−
					spleen	+	−
					lymph node	+	−
					thymus	+ #	−
JR5	7 weeks	male	HGE (deceased)	25.10.2021	rectal swab	−	+ #
					intestine	−	+ #
					lymph node	+	−
					spleen	+	−
					thymus	+ #	−
JR6	7 weeks	male	HGE (deceased)	25.10.2021	rectal swab	+ #	−
					intestine	+	−
JR7	7 weeks	female	HGE (deceased)	25.10.2021	rectal swab	+ #	+ #
					intestine	+	−
					thymus	+ #	−
					spleen	+	−
					lymph node	+ #	−
JR8	8 weeks	male	HGE (deceased)	28.10.2021	rectal swab	+ #	−
					thymus	+	−
					spleen	+ #	−
					lymph node	+	−
JR9	adult	female	asymptomatic	12.07.2021	faeces	+ #	+ #

Details of sample processing, viral metagenomics, NGS and PCR reactions, virus cultivation attempts as well as detailed descriptions of bioinformatics analyses applied in this study can be found in the supplementary Online Resource. Briefly, unprotected nucleic acids from a filtered single rectal swab sample from a 7-week-old puppy with HGE were digested with a nuclease cocktail. After total nucleic acid isolation and reverse transcription, DNA was amplified by random PCR. After library construction, the sample was run on a NextSeq (Illumina) platform and the resulted sequence data were analysed with an “*in-house*” developed bioinformatics pipeline using Kaiju v1.7.3 software and the RefSeq databases of NCBI for initial virus identification. The filtered and quality checked reads were de novo assembled using Geneious Prime Ver. 2022.1.1 (Biomatters, New Zealand).

For the determination of the complete coding sequence of CPV-2 and complete genome of CaAstV as well as for the screening of CPV-2 and CaAstV in additional enteric and tissue samples (Table 1) virus-specific primer-pairs (Table S1) and various PCR techniques such as (reverse transcription/RT/)-PCR; Taq polymerase and two oligonucleotide primer-based conventional PCR /cPCR/ and in case of CaAstV 3’/5’ rapid amplification of cDNA ends /RACE/-PCR (Roche Diagnostics, Mannheim, Germany) were used (for details see the supplementary Online Resource of this study). For confirmation of PCR positivity and pairwise sequence comparisons selected PCR products of VP2 of CPV-2 and ORF1b of CaAstV (Table 1, Table S1) were sequenced directly in both directions using the Sanger sequencing method.

For multiple sequence alignments as well as sequence and phylogenetic analyses Multiple Sequence Comparison by Log-Expectation of EMBL-EBI, Geneious Prime ver. 2022.1.1, MEGA 11 and IQ-Tree software were used, respectively.

Selected CPV-2 and CaAstV PCR positive intestinal suspensions from deceased dogs with haemorrhagic gastroenteritis were used for virus cultivation attempts on Madin-Darby canine kidney (*Canis lupus familiaris*, MDCK) cells.

## Results and discussion

**Fig. 1:**
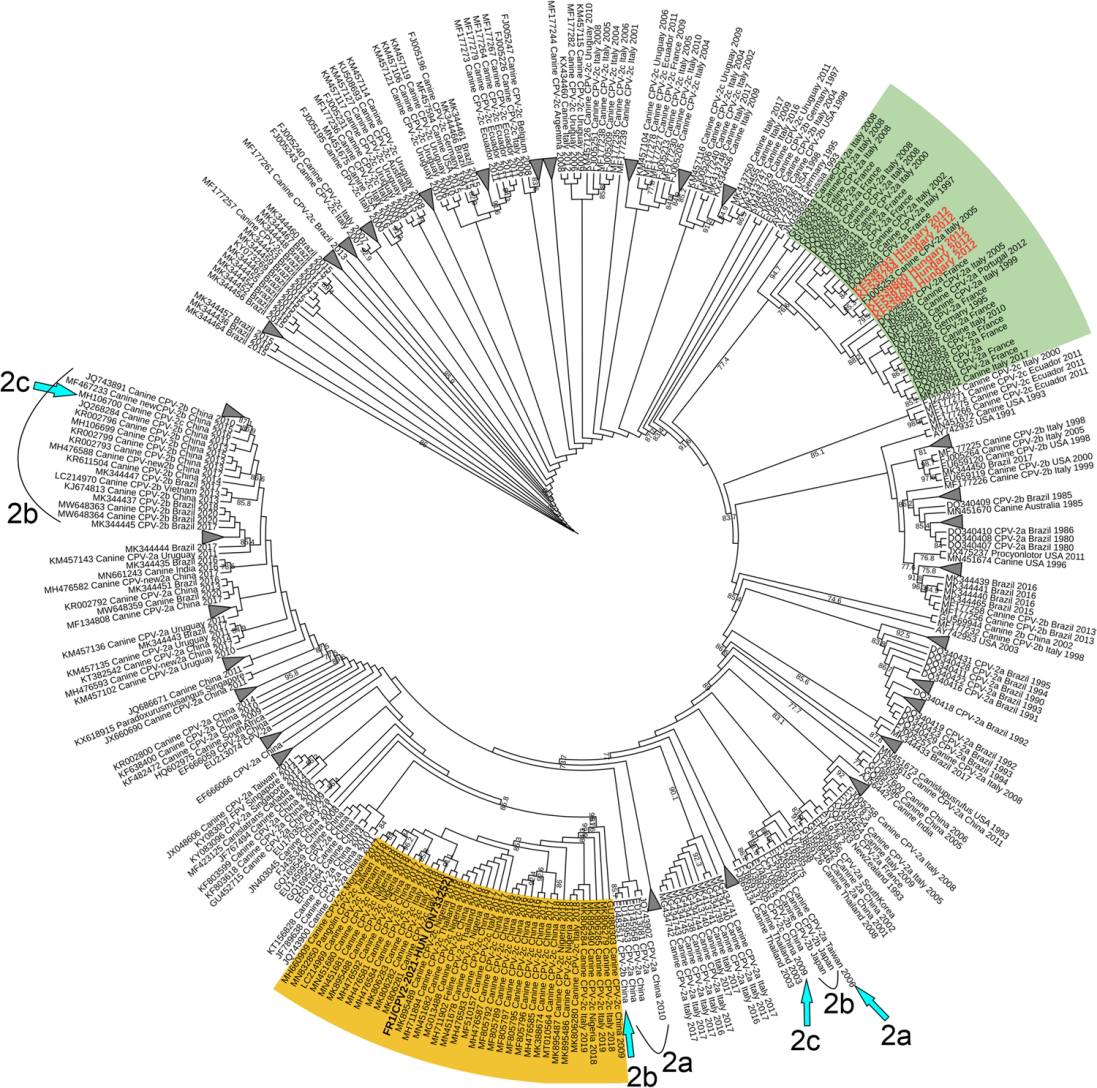
Phylogenetic tree of the study strain FR1/CPV2-2021-HUN (in black **bold**) of canine parvovirus 2 (CPV-2) and complete VP2 sequences of representative CPV-2 strains. The Maximum-likelihood tree was generated from a MUSCLE-based nucleotide alignment of *n* = 686 sequences using IQ-TREE (TVM + F + I + G4 substitution model, 1000 bootstrap replicates) and visualized by the iTOL ver. 6 web tool. Closely related sequences unrelated to our sequence collapsed because of visibility reasons. The designation of the sequences is the following: Accession number followed by the host (if available), the antigenic type (if available), the county and the year of isolation. The yellow background indicates a lineage of “Asian-origin” 2c sequences from Europe, Asia and Africa related closely to the study sequence. The green background indicates a lineage of CPV-2a sequences where the other, previously described Hungarian CPV-2a strains (written in red) are located. Cyan arrows indicate the phylogenetic locations of certain, possible VP2_426_ mutant CPV-2 strains with an antigenic type different from the closely related strains of the same cluster

From the generated 5,301,142 paired reads obtained from the NGS run of a rectal swab of a 7-week-old puppy with HGE, *n* = 4,720,025 (89.04% of the total reads) and *n* = 61,188 reads (1.15%) were classified as *Parvoviridae* (as canine parvovirus 2) and *Astroviridae* (as canine astrovirus), respectively*.* An additional *n* = 16,414 reads belonged to various phages (data not shown) while the remaining *n* = 503,515 (9.5%) reads could not be assigned to any viruses using Kaiju software. A single 4501-nucleotide (nt)-long contig generated from the *Parvoviridae* reads showed the highest (99.6%) nt sequence identity to CPV-2c isolate CPV-SH1516, (MG013488) from China. The average depth of coverage of the contig was 176,677 (ranged between 36 and 555,444). Using contig and CPV-2-specific oligonucleotide primers and cPCR-based primer walking method a 5016-nt-long, nearly complete genome of the CPV-2 strain FR1/CPV2-2021-HUN (ON733252) was determined which includes the complete coding sequences (CDS) of NS1/NS2 and VP1/VP2. Due to the presence of multiple repeated/duplicated sequence motifs at both 3’ and 5’ ends – like in other CPV-2 viruses (Reed et al. 1988) – the complete genome could not be determined by available PCR methods. The full length, 668-aa-long NS1 of the study strain showed the highest aa sequence identity (99.85%, only a single aa difference in N558S) to a CPV-2c strain IZSSI_PA5632/19 (MK806285) from Italy. The major, 584-aa-long VP2 capsid protein also showed the highest sequence identity (99.83%, only a single aa difference in D426E) to a CPV-2c strain IZSSI_PA31342/18 (MK806281) from Italy (Mira et al. 2019). Phylogenetically the study strain also clustered together with CPV-2c strains from Italy, Nigeria and Asia identified mostly in the last five years and clearly separated from the previously detected Hungarian CPV-2 strains in the VP2 tree (Fig. 1) which strongly indicates the close phylogenetic relationship of the study strain and the recently emerged “Asian-origin” 2c strains*.* Furthermore, all generally known aa mutations in NS1, NS2 and in VP2 which are known to be characteristics of “Asian-origin” CPV-2c strains were also present in the study strain. But interestingly, because the antigenic typing of CPV-2 strains is based on the presence of N, D or E aa-s in position 426 of VP2 the study strain is a 2b-type antigenic variant (426D) rather than 2c (426E) as the closest strains. This aa change was caused by a GAA_1278_ → GAT_1278_ non-synonymous mutation in VP2 which was also confirmed by Sanger sequencing.

CPV-2 was detectable by cPCR in 8 out of 9 available rectal swabs and 32 out of 36 organ samples from the gastrointestinal tract (intestine: 7/8 positive), immune system (thymus: 7/7 positive, spleen: 8/8 positive, bone marrow: 2/4 positive, lymph nodes: 8/8 positive) and liver: 0/1 positive (Table 1) of symptomatic animals suggested the presence of disseminated CPV-2 infection similar to found in non-vaccinated cases (Decaro and Buonavoglia 2012). Partial 1589-nt-long VP2 sequences of the selected strains – determined by cPCR and Sanger-sequencing Table 1 – were completely identical to each other and all contained the GAA_1278_ → GAT_1278_ mutation identified in the study strain of FR1/CPV2-2021-HUN (data not shown), indicating that this non-synonymous mutation was fixed of this field strain circulating in this colony. Furthermore, the same CPV-2 was detectable from a faecal sample of an asymptomatic adult bitch, a mother of deceased puppies with the signs of HGE suggesting the role of adults in spreading this CPV-2 strain. The puppies were vaccinated against CPV-2 either by Canigen® DHPPi or Primodog™ vaccines. These vaccine strains were checked by cPCR and Sanger sequencing. There were 22 nt (6 aa) differences identified in the partial, 1589 nt-long VP2 sequences between the study strain of FR1/CPV2-2021-HUN and Canigen, or Primodog CPV-2b vaccine strains, respectively (data not shown) suggesting that the identified field strain is most likely not a vaccine revertant mutant. Based on the continuous spreading of “Asian-origin” CPV-2 strains in nearby countries like Italy and Romania (Mira et al. 2019; Novosel et al. 2019; Balboni et al. 2021) the appearance of an “Asian-origin” CPV-2 in Hungary is not unexpected, although to our current knowledge the fixed non-synonymous E426D mutation in VP2 in a field strain of this lineage is quite unusual. Based on previous phylogenetic analyses of CPV-2 VP2 sequences similar mutations affecting this site (VP2_426_) – and therefore causing a change of the antigenic variant type – are rare but not unprecedented (Voorhees et al. 2019). The presence of a few possible VP2_426_ mutant CPV-2 strains with an antigenic type different from the closely related strains of the same cluster are also observable in our VP2 phylogenetic tree (Fig. 1).

Furthermore, sequence data from CPV-2 strains circulating in Hungary was available only between 2010 and 2014, when 2a type was solely identified (Demeter et al. 2010; Cságola et al. 2014) (Fig. 1). Because of the presence of “Asian-origin” CPV-2 in the investigated breed, further epidemiological investigations are needed to explore the dominant CPV-2 strains circulating currently in Hungary. After eight passages no visible cytopathic effect (CPE) was observable in Madin-Darby canine kidney (MDCK) cell line inoculated with CPV-2 and CaAstV PCR positive intestinal suspensions from deceased dogs with haemorrhagic gastroenteritis. The lack of visible CPE indicating that neither CPV-2 nor CaAstV were cultivable in the applied MDCK line.

**Fig. 2:**
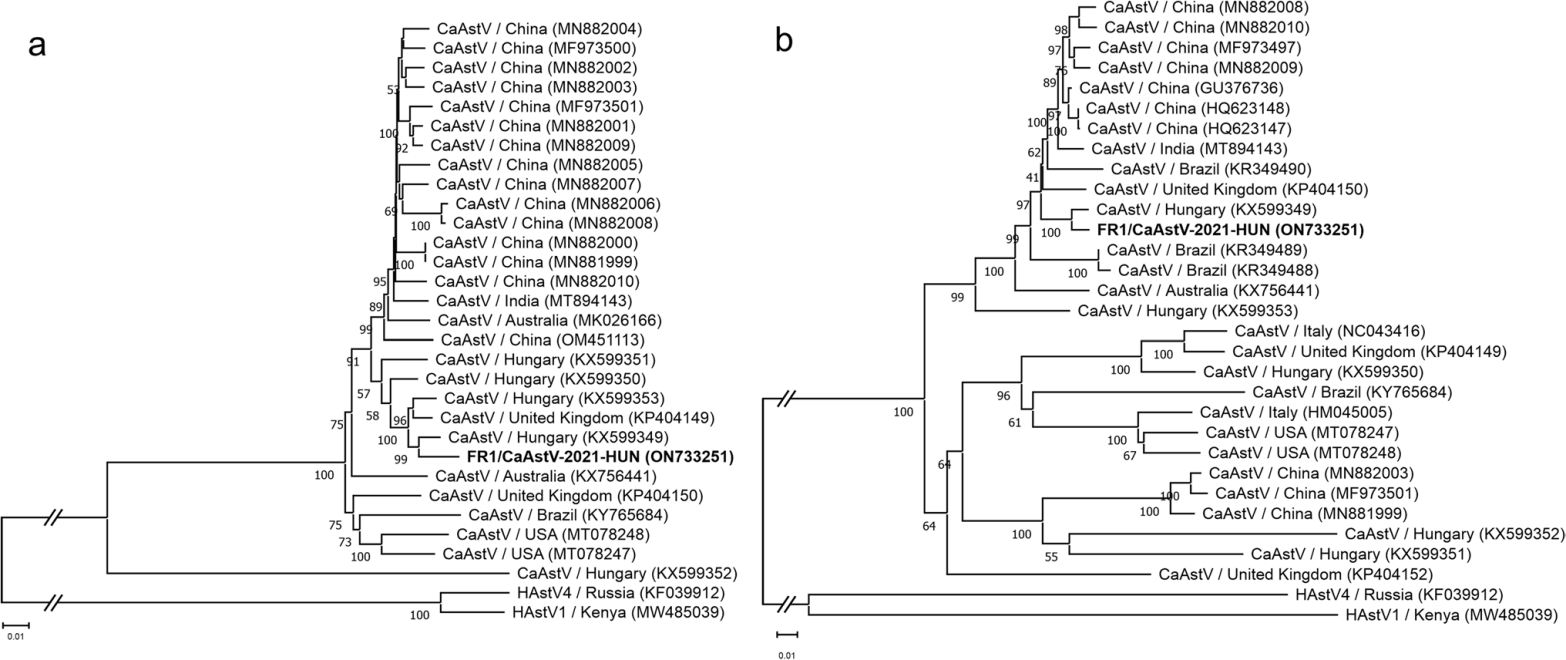
Phylogenetic trees complete ORF1ab (**a**) and ORF2 sequences (**b**) of the study strain FR1/CaAstV-2021-HUN (in **bold**) and canine astroviruses (CaAstVs) with selected human AstVs (HAstV) as outgroups. The Neighbor-Joining trees were generated from MUSCLE-based nucleotide alignments using the Jukes-Cantor model and bootstrapped with 1000 replicates by MEGA ver. 11. The scale bars indicate the number of nucleotide substitutions per site. The designation of the sequences is the following: virus type/country of origin and accession number in brackets

Besides CPV-2, a single 5484-nt-long CaAstV-related contig was also generated from the *n* = 61,188 astrovirus reads by Geneious R11 software. The average depth of coverage of the contig was 2089.3 (ranged between 1 and 4,991). Using contig and CaAstV sequence specific primers (Table S1) and various PCR techniques (RT-PCR, 3’/5’ RACE-PCR) the 6569-nt-long complete genome of FR1/CaAstV-2021-HUN (ON733251) was determined. The 4157-nt-long ORF1ab and the 2475-nt-long ORF2 showed 97.48% and 98.06% nt identity to the corresponding genomic regions of CaAstV strain HUN/2012/2 (KX599349) as the most similar sequence identified by BLASTn search. The closest strain was originally detected in a diarrhoeic, 5-week-old dog in Hungary, in 2012 (Mihalov-Kovács et al. 2017). The phylogenetic analyses of the ORF1ab and ORF2 sequences of CaAstVs also showed close relationship between the FR1/CaAstV-2021-HUN and HUN/2012/2 of Hungary (Fig. 2).

CaAstV was identified by RT-PCR only from 6 of the 8 available intestinal, and 7 of the 9 investigated rectal swab samples of diarrhoeic dogs as well as a single available faecal sample of a mother bitch (Table 1). RT-PCR positivity was confirmed by Sanger-sequencing in selected samples (Table 1). All the determined 330-nt-long partial ORF1ab sequences show complete identity to the corresponding genome part of FR1/CaAstV-2021-HUN (data not shown). Our results could indicate the continuous presence and circulation of CaAstV in the colony. No other organs were RT-PCR positive for CaAstV (Table 1) suggesting that at least in these cases CaAstV infection was restricted to the gastrointestinal tract in symptomatic dogs without detectable systemic dissemination. Certain strains of CaAstVs were also described previously in diarrhoeic cases, sometimes as a co-infecting agent with CPV-2 (Caddy and Goodfellow 2015; Bhatta et al. 2019).

In summary, the identified field strain of “Asian-origin” CPV-2 was able to cause disseminated infections and – together with CaAstV – recurrent outbreaks of acute HGE with a high mortality rate among puppies vaccinated against CPV-2. The reason(s) behind the outbreak despite of the vaccination could be due to the highly contagious and possibly rapidly replicating nature of the identified wild, field CPV-2 strain which is potentially passing from the infected mother bitch to the puppies before (or around) the time of administration of the first dose of CPV-2 vaccine. Furthermore, (i) the insufficient time for the development of vaccine-induced protective antibodies, or (ii) the insufficient level of vaccine protection (vaccine-escape?) and/or (iii) the presence of interfering maternal antibodies in 3-weeks old puppies and/or (iv) the presence of other gastroenteric viruses (e.g., CaAstV or other undetectable/not investigated agents) could also contribute to the disease and resulting deaths. Purebreds were previously found to be more susceptible to CPV-2 infections than native dogs and “Asian-origin” CPV-2c strains could cause more severe infections than the other types (Decaro and Buonavoglia 2012; Qi et al. 2020). Nevertheless, further comparative studies are necessary to confirm the role of the point mutation causing the change in antigenic type 2c to 2b of this “Asian-origin” CPV-2 in the context of the presently used CVP-2 vaccines efficacy and/or the role of CaAstV co-infection in the development and/or severity of (haemorrhagic) gastroenteritis among dogs vaccinated against CPV-2. CPV-2 variants circulating among vaccinated dogs should also be monitored more vigorously considering that this virus has the capacity to infect not just dogs and other wild carnivores but – as described recently– swine as well (Decaro and Buonavoglia 2012; Temeeyasen et al. 2022).

.

## Supplementary Information

Below is the link to the electronic supplementary material.

Supplementary file1 (DOC 83 KB)

## Data Availability

Sequence data of canine parvovirus 2 strain FR1/CPV2-2021-HUN and canine astrovirus strain FR1/CaAstV-2021-HUN were uploaded to the GenBank database under accession numbers of ON733251 and ON733252. The datasets generated during the current study are available from the corresponding author on reasonable request.
