# Supplementary material for: Unusual “Asian-origin” 2c to 2b point mutant canine parvovirus (Parvoviridae) and canine astrovirus (Astroviridae) co-infection detected in vaccinated dogs with an outbreak of severe haemorrhagic gastroenteritis with high mortality rate in Hungary
Source: Vet Res Commun. 2022 Sep 21;46(4):1355–61. doi: 10.1007/s11259-022-09997-2 (PMC9490711; doi:10.1007/s11259-022-09997-2)
Supplement: Supplementary file 1 — Supplementary file1 (DOC 83 KB) [file 11259_2022_9997_MOESM1_ESM.doc]

***Veterinary Research Communications***

**Online Resource**

**Unusual “Asian-origin” 2c to 2b point mutant canine parvovirus (*Parvoviridae*) and canine astrovirus (*Astroviridae*) co-infection detected in vaccinated dogs with an outbreak of severe haemorrhagic gastroenteritis with high mortality rate in Hungary**

Ákos Boros^1^*, Mihály Albert^2^, Péter Urbán^3^, Róbert Herczeg^3^, Gábor Gáspár^1^, Attila Cságola^2^, Péter Pankovics^1^, Attila Gyenesei^3^, Gábor Reuter^1^

^1^ Department of Medical Microbiology and Immunology, Medical School, University of Pécs, Pécs, Hungary,

^2^ Ceva Phylaxia Ltd. Budapest, Hungary;

^3^ Szentágothai Research Centre, Bioinformatics Research Group, Genomics and Bioinformatics Core Facility, University of Pécs, Pécs, Hungary.

***** Correspondence: [boros.akos@pte.hu](mailto:boros.akos@pte.hu) ; Tel.: +36 72 536-251

***Sample pre-treatment for next-generation sequencing***

The single anal swab sample from a 7-week-old puppy with HGE was re-suspended in 500µl 0.1M phosphate-buffered saline (PBS) and filtered with 0.45µm sterile filter (Merck-Millipore, Burlington, USA). The free nucleic acids (NAs) were digested with 14U Turbo DNase (Ambion, Life Technologies, Grand Island, NY, USA), 3U Baseline-ZERO (Epicentre, Chicago, IL, USA) and 20U RNase One (Promega, Madison, WI, USA) in 1×DNase buffer (Ambion, Life Technologies, Grand Island, NY, USA) and incubated at 37^o^C for 1.5 h (Li et al., 2015). Viral nucleic acids were purified using Quick-RNA Viral Kit (Zymo Research, Irvine, USA) according to the manufacturer’s instructions but without the DNAse-treatment step. cDNA synthesis was performed on 10µl of NAs using 200U of MAXIMA-RT enzyme (Thermo Fisher, Waltham, USA) and 100 pmol of adapter random hexamer primer (5’–GCGGCCGCCACCAATTTAAATNNNNNN–3’) according to the protocol provided by the manufacturer. Second strand DNA was synthesized using Klenow-fragment (New England Biolabs, Ipswich, USA) and random-hexamer according to the manufacturer’s instructions.

One µl of the RT-products were amplified by random PCR using 2.5µM adapter primer (GCGGCCGCCACCAATTTAAAT) with 1U DreamTaq DNA polymerase (Thermo Fisher, Waltham, USA) 2.0mM MgCl_2_, 0.5mM dNTPs, in a reaction volume of 25µl. Temperature cycling was performed as follows: 1 cycle of 95^o^C for 1min, 39 cycles of denaturing at 95^o^C for 20 s, 58^o^C for 20 s, 72^o^C for 1.5 min followed by an additional extension step for 10 min at 72^o^C. The PCR-products were purified using a GeneJET PCR purification kit (Thermo Fisher, Waltham, USA) according to the protocol provided by the manufacturer.

***Library preparation and next-generation sequencing***

The library for Illumina sequencing was prepared using Swift 2S Turbo DNA Library Kit (Swift Biosciences, Ann Arbor, MI, USA). Briefly, 100 ng PCR-products were fragmented, end prepped and adapter-ligated. Then magnetic beads size selection was performed to select 250-300 bp insert size fragments. Finally, the library was amplified according to the manufacturer’s instructions. The quality of the library was checked on the 4200 TapeSation System using D1000 Screen Tape (Agilent Technologies, Palo Alto, CA, USA), and the quantity was measured on Qubit 3.0 (Thermo Fisher, Waltham, MA, USA). Illumina sequencing was performed on NovaSeq 6000 instrument (Illumina, San Diego, CA, USA) with a 2x151 run configuration.

***Bioinformatics analyses of the NGS data***

Obtained raw reads were quality checked (FastQC v0.11.9) and adapter trimmed with fastp (v0.21.0; phred score >= 25, and length > 40). Kaiju v1.7.3 (Menzel et al. 2016) was used for taxonomic classification based on the viruses (only viruses from the NCBI RefSeq database) and the nr (subset of NCBI BLAST nr database containing all proteins belonging to Archaea, Bacteria and Viruses) databases which are available in Kaiju. Paired end reads were assigned via the following basic parameters: run mode: greedy; allowed mismatch: 3; minimum match length: 11; e-value: 0.01. The filtered and quality checked reads were *de novo* assembled using Geneious Prime Ver. 2022.1.1. The generated contigs were identified by BLASTn/x searches.

***PCR-based screening and genome acquisition reactions***

Prior to total nucleic acid (NAs) isolations anal swabs were re-suspended in 500µl sterile 0.1M PBS and ~40v/v% suspension was created from a faecal sample with the use of sterile 0.1M PBS. The faecal suspension was centrifuged at 10.000 rpm for 5 min and the supernatant was used for NA isolation. Total NA was extracted from 150µl of the prepared samples by the use of Quick-RNA Viral Kit (Zymo Research, Irvine, USA) according to the manufacturer’s instructions but without DNAse-treatment step.

50-100 mg of tissue samples were homogenized manually in the lysis buffer of the applied Absolutely RNA Miniprep Kit (Agilent Technologies, Santa Clara, USA) using Potter-Elvehjem tissue grinder (Sigma-Aldrich, St. Louis, USA) and total NAs were extracted according to the protocol provided by the manufacturer but omitting the use of DNAse-digestion. For genome determination and screening reactions virus-specific oligonucleotide primers (Table S1) and various PCR techniques such as reverse transcription (RT)-PCR; Taq polymerase and two oligonucleotide primer-based conventional PCR /cPCR/, as well as in case of CaAstV 5’/3’ rapid amplification of cDNA ends (RACE) methods (Roche Diagnostics, Mannheim, Germany) were used (*Boros et al., 2011*).

In brief, Maxima reverse transcriptase enzyme (Thermo-Fisher Waltham, MA) and terminal deoxynucleotidyl transferase (TdT) enzyme with dATP (Thermo-Fisher Waltham, MA) were used for reverse transcription and cDNA tagging (5’RACE only) reactions, respectively. For the 3’/5’ PCR reactions sequence-specific reverse (5’RACE) or forward (3’RACE) primers and an anchored oligo dT-adapter primer (Table S1) was used in a conventional PCR reaction. Temperature cycling was performed as follows: 1 cycle of 95^o^C for 1min, 39 cycles of denaturing at 95^o^C for 20 s, 50^o^C for 20 s, 72^o^C for 1.5 min followed by an additional extension step for 10 min at 72^o^C. The PCR-products were purified using a GeneJET PCR purification kit (Thermo-Fisher, Waltham, USA) according to the protocol provided by the manufacturer.

The conditions and reagents used in the genome acquisition and screening PCR reactions were the same as described previously (*Boros et al., 2012; László et al., 2021*). Selected PCR-products were sequenced directly in both directions using BigDye Terminator v1.1 Cycle Sequencing Kit (Thermo Fisher, Waltham, USA) and run on an ABI 3500 Genetic Analyzer (Applied Biosystems, Hitachi, Tokyo, Japan). For the analyses of sequence data Chromas Ver. 2.6.6 and Geneious Prime ver. 2022.1.1 (Biomatters, New Zealand) software were used.

***In silico sequence and phylogenetic analyses***

For CPV-2 VP2 nucleotide phylogenetic tree, beside the study strain of CPV-2 FR1/CPV2-2021-HUN additional n=685 complete VP2 sequences (selected by the description of de Oliveira Santana at al., 2022) were downloaded from GenBank. For CaAstV ORF1ab and ORF2 phylogenetic trees n=28 of the most closely related sequences identified by BLASTn searches of both genomic regions (ORF1ab, ORF2) as well as n=2 human astrovirus sequences as outgroups were downloaded from the GenBank database.

Multiple sequence alignments used for phylogenetic analyses and sequence comparisons were generated by the online platform of Multiple Sequence Comparison by Log-Expectation of EMBL-EBI. GeneDoc software ver. 2.7 and Geneious Prime ver. 2022.1.1 (Biomatters, New Zealand) were used for the assembly of generated sequences, genome analyses as well as pairwise identity calculations. Phylogenetic trees were created from the nucleotide sequence alignments using either MEGA 11 (CaAstV-ORF1ab and ORF2 trees) or IQ-Tree software (VP2 tree of CPV-2) and visualized by the iTOL ver. 6 web tool (Trifinopoulos et al., 2016; Letunic & Bork 2021; Tamura et al., 2021). The applied setup of the trees was described separately in the figure legends of each phylogenetic trees.

***Cell culture and virus inoculation***

Madin-Darby canine kidney cells (*Canis lupus familiaris*, MDCK), were cultured at 37°C in a 5% CO_2_ humidified atmosphere in Dulbecco's Modified Eagle Medium:Nutrient Mixture F-12 (DMEM-F12, Gibco/Thermo Fisher, Waltham, MA, USA) supplemented with 10% foetal calf serum (FCS) and 10mg/L gentamycin antibiotic solution (Sigma-Aldrich, St. Louis, MI, USA) CPV-2 and CaAstV PCR positive intestinal suspensions from deceased dogs with haemorrhagic gastroenteritis used for inoculations were first centrifuged (10.000 x rpm, 10 min) then the supernatants were inoculated on ≈80% confluent cells grown in sterile 25 cm^2^ cell culture flasks. The inoculums were incubated with the cells for 60 min at 37°C followed by the addition of a fresh medium. After 4 days of incubation, the cell cultures were assessed for cytopathic effects (CPE). Following repeated freeze-thawing and centrifugation (1000 x g for 10 min) the culture supernatants were transferred to fresh cell cultures (passages). Up to eight passages with similar steps were applied. Viral growth was monitored by inspecting the presence of CPE.

| **Targeted genome region** | **Primer name*** | **5' - 3' sequence** | **PCR Product length (bp)** | **Reaction type** |
| --- | --- | --- | --- | --- |
| VP2 (CPV-2) | CaParV-2698-F | AAG TAA ACC ACC ACC TCA TAT | 1831/960 | screening |
|  | CaParV-4529-R | TTC TAG GTG CTA GTT GAG ATT |  |  |
|  | CaParV-3658-R | TAG AAA TGG TGG TAA GCC CAA |  |  |
| 3' end (CPV-2) | CaParV-2-F | TCT TTA GAA CCA ACT GAC CAA G | 748/310 | genome sequencing |
|  | CaParV-312-R | ACT CCC TCC ATA ACT TCC TCA |  |  |
|  | CaParV-750-R | ACC AAC CAT CTA CTC CAA TAC A |  |  |
| 5' end (CPV-2) | CaParV-5016-R | CGA GGC CAT TTA GTT TTA AAT G | 610 | genome sequencing |
|  | CaParV-4407-F | CCT CTC ATA CTT GGA ATC CA |  |  |
| ORF1a (CaAstV) | CaAstV-281-R3 | CCG TTT TCA AGA CCA CCG GTT | 311/288@ | genome sequencing |
|  | CaAstV-311-R2 | TCA CCA TCA TGA ACA ACA GTA |  |  |
|  | CaAstV-366-R1 | GAG CAC TCT GAG CAG TCC TT |  |  |
| ORF1b (CaAstV) | CaAstV-3876-R | GCT TAA CCC ACA TTC CAA A | 330 | screening |
|  | CaAstV-3547-F | AAC AGA GGA GGA AAT ACC AA |  |  |
| ORF2 (CaAstV) | CaAstV-4222-F | CAG GCA AAG ATG TTA CCG TTG A | 1000 | genome sequencing |
|  | CaAstV-5226-R | GGG AAC AAT GAC TGA TGC TGC |  |  |
|  | CaAstV-5179-F | TGG CAG ATC GCT TCG ACT GCA | 1400/805# | genome sequencing |
|  | CaAstV-5782-F | TCA CAA CAT AAA GTC TCT AGC A |  |  |
|  | anchored-oligo dT-adapter | GACACGCGTATCGATGTCGACT_12_V |  |  |

**TableS1:** List and characteristics of oligonucleotide primers and reactions used in this study. CPV-2: canine parvovirus 2, CaAstV: canine astrovirus. * numbers in the names of the primers refer to the first or last positions of binding sites of the forward or reverse primers, respectively on reference complete genomes of CPV-N (M19296) of CPV or HUN/2012/2 (KX599349) of CaAstV. # product lengths refer to the sizes of PCR products in 3' RACE PCR reactions where anchored oligo dT-adapter primer was used as reverse primer. @: product lengths refer to the sizes of PCR products in 5' RACE PCR reactions where anchored oligo dT-adapter primer was used as a forward primer.

**References**

Boros Á, Pankovics P, Simmonds P, Reuter G (2011) Novel positive-sense, single-stranded RNA (+ssRNA) virus with di-cistronic genome from intestinal content of freshwater carp (Cyprinus carpio). PLoS One, 6, doi:10.1371/journal.pone.0029145

Boros Á, Pankovics P, Knowles NJ, Reuter G (2012) Natural interspecies recombinant bovine/porcine enterovirus in sheep. J Gen Virol 93, 1941–1951, doi:10.1099/vir.0.041335-0

László Z, Pankovics, P, Reuter G, Cságola A, Bálint Á, Albert M, Boros Á (2021) Multiple types of novel enteric bopiviruses (Picornaviridae) with the possibility of interspecies transmission identified from cloven-hoofed domestic livestock (ovine, caprine and bovine) in Hungary. Viruses, 13(1), 66.

Tamura K, Stecher G, Kumar S (2021) MEGA11: molecular evolutionary genetics analysis version 11. Mol Biol Evol 25;38(7):3022-3027. doi: 10.1093/molbev/msab120.

Trifinopoulos J, Nguyen LT, von Haeseler A, Minh BQ (2016) W-IQ-TREE: a fast online phylogenetic tool for maximum likelihood analysis. Nucleic Acids Res 8;44(W1):W232-5. doi: 10.1093/nar/gkw256

Letunic I, Bork P (2021) Interactive Tree Of Life (iTOL) v5: an online tool for phylogenetic tree display and annotation. Nucleic Acids Res 2;49(W1):W293-W296. doi: 10.1093/nar/gkab301.

Li L, Deng X, Mee ET, Collot-Teixeira S, Anderson R, Schepelmann S et al (2015) Comparing viral metagenomics methods using a highly multiplexed human viral pathogens reagent. J Virol Methods 213:139-46. doi: 10.1016/j.jviromet.2014.12.002.

Menzel P, Ng KL, Krogh A (2016) Fast and sensitive taxonomic classification for metagenomics with Kaiju. Nat Commun 13;7:11257. doi: 10.1038/ncomms11257.

de Oliveira Santana W, Silveira VP, Wolf, JM, Kipper D, Echeverrigaray S, Canal CW et al (2022) Molecular phylogenetic assessment of the canine parvovirus 2 worldwide and analysis of the genetic diversity and temporal spreading in Brazil. Infect Gene3t Evol. 98, 105225.
